# Supplementary material for: Impact of Sex on Mortality in Patients Undergoing Surgical Aortic Valve Replacement
Source: J Pers Med. 2022 Jul 24;12(8):1203. doi: 10.3390/jpm12081203 (PMC9331870; doi:10.3390/jpm12081203)
Supplement: Supplementary file 1 [file jpm-12-01203-s001.zip › jpm-1818390-supplementary.pdf]

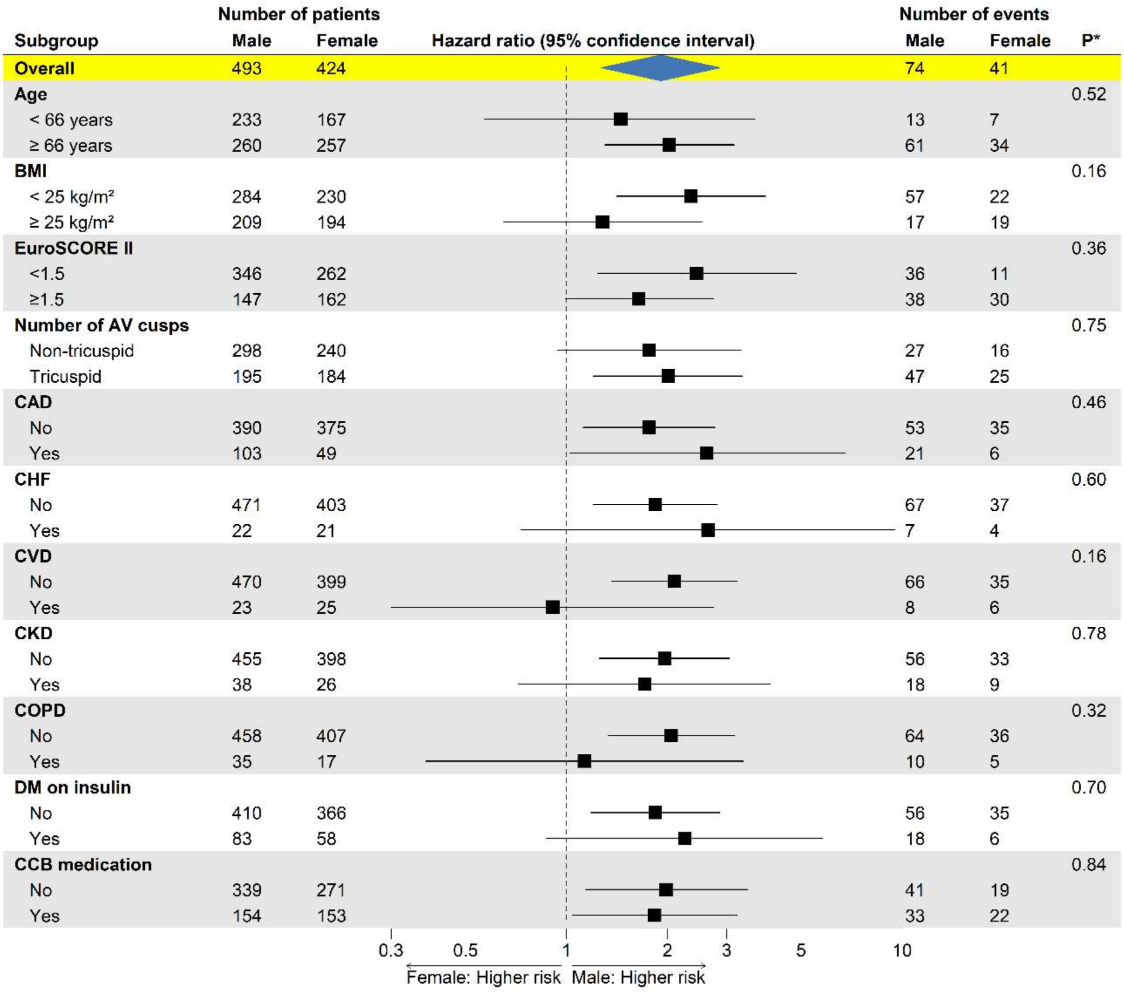

Figure S1. Forest plot of subgroup analysis stratified by sex. BMI, body mass index; EuroSCORE II, European system for cardiac operative risk evaluation II; CAD, coronary artery disease; CHF, congestive heart failure; CVD, cerebrovascular disease; CKD, chronic kidney disease; COPD, chronic obstructive pulmonary disease; DM, diabetes mellitus; CCB, calcium channel blocker. P*: P value for interaction.
